# Applicability of the Test of Variables of Attention – T.O.V.A in Brazilian adults

**DOI:** 10.1590/1980-57642018dn12-040009

**Published:** 2018

**Authors:** Cláudia M. Memória, Henrique C.S. Muela, Natália C. Moraes, Valéria A. Costa-Hong, Michel F. Machado, Ricardo Nitrini, Luiz A. Bortolotto, Monica S. Yassuda

**Affiliations:** 1 Department of Neurology, University of São Paulo, São Paulo, SP, Brazil; 2 Heart Institute (Incor), Hypertension Unit, University of São Paulo, São Paulo, SP, Brazil; 3 Department of Physiology, Faculty of Medicine, Agostinho Neto University, Luanda, Angola.

**Keywords:** attention, computerized evaluation, adults, elderly, atenção, avaliação informatizada, adultos, idosos

## Abstract

The functioning of attention is complex, a primordial function in several cognitive processes and of great interest to neuropsychology. The Test of Variables of Attention (T.O.V.A) is a continuous computerized performance test that evaluates some attention components such as response time to a stimulus and errors due to inattention and impulsivity.

**Objective:**

1) To evaluate the applicability of T.O.V.A in Brazilian adults; 2) To analyze the differences in performance between genders, age ranges, and levels of education; 3) To examine the association between T.O.V.A variables and other attention and cognitive screening tests.

**Methods:**

The T.O.V.A was applied to 63 healthy adults (24 to 78 years of age) who also underwent the Mini-Mental State Examination (MMSE), Montreal Cognitive Assessment (MoCA), Digit Span and Digit Symbol (Wechsler Intelligence Scale for Adults – WAIS-III) and the Trail Making Test.

**Results:**

the T.O.V.A was little influenced by age or education, but was influenced by gender. The correlations between some T.O.V.A variables and the Digit Symbol and Trail Making test were weak (r-values between 0.2 and 0.4), but significant (p<0.05). There was no correlation with the Digit Span test.

**Conclusion:**

The T.O.V.A showed good applicability and proved adequate for evaluating attentional processes in adults.

## RESUMO

O funcionamento da atenção é complexo, função primordial em diversos processos cognitivos e de grande interesse para a neuropsicologia. O Teste de Variáveis de Atenção (T.O.V.A) é um teste computadorizado de desempenho contínuo que avalia alguns componentes de atenção, como tempo de resposta a um estímulo e erros por desatenção e impulsividade.

**Objetivo:**

1) Avaliar a aplicabilidade do T.O.V.A em adultos brasileiros; 2) Analisar as diferenças de desempenho entre os gêneros, faixas etárias e níveis de escolaridade; 3) Examinar a associação entre as variáveis T.O.V.A e outros testes de atenção e triagem cognitiva.

**Métodos:**

O TOVA foi aplicado a 63 adultos saudáveis (24 a 78 anos) submetidos ao Mini-Exame do Estado Mental (MEEM), Montreal Cognitive Assessment (MoCA), Digit Span e Digit Symbol (Wechsler Intelligence Scale for Adults – WAIS-III) e o Trail Making Test.

**Resultados:**

T.O.V.A teve pouca influência da idade e escolaridade, mas foi influenciado pelo gênero. As correlações entre algumas variáveis T.O.V.A e o símbolo Digit e o teste Trail Making foram fracas (valores de r entre 0,2 e 0,4), mas significativas (p <0,05). Não houve correlação com o teste Digit Span.

**Conclusão:**

T.O.V.A apresentou boa aplicabilidade e foi adequado para avaliar os processos de atenção em adultos.

In most models, attention is portrayed as a complex system that allows the individual to filter relevant and irrelevant information, maintain and process mental representations, and monitor and modulate responses to stimuli. Attention, therefore, usually refers to a multifactorial set of processes that goes beyond the simple ability to encode information. Attention includes several processes, such as sensory selection (filter, focus, alternation), response selection (response intention, initiative and inhibition, active change and executive control), and attentional capacity (effort, sustained performance, alertness).^1^ Attentional deficits can therefore affect one or more of these processes and can be classified according to their particularities.

Frequently, the Digit Span,^2^^,^ 3 a subtest of the Wechsler Scale is used to assess auditory-verbal attention. However, it is a short-term and uninformative task on fatigue, inhibition and attentional oscillations throughout the execution time. To take into account these aspects, *Continuous Performance Tests* (*CPT,*) such as the *Test of Variables of Attention (T.O.V.A)* 4 would be more appropriate for examining sustained attention and providing data on the other parameters.

The T.O.V.A is a 21-minute computerized test in which a simple geometric stimulus is used to measure the response to visual stimuli. It can be used in serial evaluations and assists in the detection of attention disorders. The test involves the presentation of two distinct situations: the display of a target stimulus (a small square in the upper part presented within a larger square), upon which the individual must press the microswitch, and the presence of a different stimulus (a small square in the lower part within a larger square) for which the subject must not press the microswitch. Being computerized battery, it offers advantages such as ease of application and handling, less influence of the examiner, automatic generation of report with results, and accuracy in recording of reaction time (±1 millisecond).

This test can also be used to monitor the response to drug treatment because it has negligible test-retest effects.^4^ US standards for the T.O.V.A are based on data from 1,596 individuals aged 4 to 80 years, with a shorter version of the test available for pre-school children.^4^ However, few studies have been performed on the T.O.V.A in adults.

Additionally, the test is expected to be little influenced by education because the instructions and stimuli involved in the test are simple. The test has been mainly used as a clinical outcome variable for interventions aimed at reducing symptoms in ADHD^5^ while few studies have used it as a diagnostic tool for attention deficit in adults.^6^^,^ 7

No studies conducted in Brazil on the T.O.V.A were found. Thus, the objectives of the present study were: 1) to evaluate the applicability of the T.O.V.A in Brazilian adults without chronic diseases; 2) to investigate whether the T.O.V.A scores vary between the sexes, age groups and education; and 3) to investigate the association between the scores generated by the T.O.V.A and other attention tests.

## METHODS

### Participants

The sample consisted of 63 adults with a mean age of 52.05 (±14.43) years and a mean educational level of 13.13 (±4) years. Participants were individuals included in the CHEST-BR study,^8^ in which subjects without cardiovascular disease underwent a complete clinical evaluation, laboratory tests, ergometric test and echocardiogram. Subjects were included in the study if no change was evident on these assessments. Participants who were hypertensive, diabetic, smokers or in chronic use of alcohol were excluded.

### Instruments

#### Test of Variables of Attention (T.O.V.A)

The test starts with a three-minute training phase,^4^ where the participant is instructed to press the microswitch in the presence of a target stimulus.

The participant is not informed about the structure of the test and usually does not know that the test consists of two parts: during the first half of the test (lasting 10 minutes), the target appears in 22.5% of the trials. During the second half (same length of time), the target appears in 77.5% of the trials. The increasing frequency of the target implies that the examinee must respond more quickly. When the subject does not respond to the target, this response is called an error of omission and is a measure of inattention; and when the examinee responds to a non-target, the response is called a *commission error* and considered a measure of impulsiveness.^4^^,^ 9 Thus, the first half of the test is designed to maximize the demand for sustained attention (and induces omission errors), while the second half calls for inhibitory control (and induces commission errors).^10^

In addition to the primary variables, the T.O.V.A generates secondary variables that include variability in response time (consistency), response time, the D-*prime*, and an attentional performance score. The measures are outlined below:

- Omission Errors: considered a measure of inattention. The subject does not respond to the predetermined target; that is, the subject fails to press the T.O.V.A microswitch when the target is displayed. The omission score is calculated as the ratio between the number of correct responses to the target and the subtraction of the actual number of targets displayed from the number of anticipatory responses to the targets.
- Commission Errors: considered a measure of impulsivity or behavioral disinhibition. The subject is unable to inhibit the response and incorrectly responds to a non-target stimulus, pressing the button when a non-target is displayed. The commission score is calculated as the ratio between the number of incorrect responses to the non-targets and the subtraction of the actual number of non-targets presented from the number of anticipatory responses made to non-target stimuli.
- Response time variability: a measure of time differences for correct responses given by the subject, denoting the consistency in the speed of correct responses. The faster the subject, the lower the variability.
- Response time: the average time a subject takes to respond correctly to a target by pressing the microswitch from the moment the target is presented. The response time score is the average of the response times (correct responses), calculated as the sum of all times divided by the number of correct responses to the target, expressed in milliseconds (ms) for each quartile, half and total time of the test.
- D-prime: a response sensitivity score that reflects the ratio between the rate of correct responses and the rate of “false alarm”. It is considered a measure of decrease in performance over time, that is, the rate of decline of performance throughout the task. This measure is derived from the Signal Detection Theory and helps to distinguish individuals without impairment from those diagnosed with attention disorders.^11^ The score reflects the accuracy in discriminating between target (signal) and non-target (noise) and is interpreted as a measure of perceptual sensitivity.
- Attentional Performance Index: the result of the comparison of the performances of the studied subjects on the T.O.V.A versus an American sample identified as having ADHD. Positive indices are suggestive of no attention impairment. The formula used to derive the score is as follows: response time in z score (1st half of the test) + D-prime in z score (2nd half of the test) + variability in response time in z score (total). In subjects with ADHD, the index is expected to be negative, although not specific to this disorder because attentional impairments may be present in various conditions.^4^

The T.O.V.A has some criteria invalidating the test run: response time or variability equal to zero in any quartile of the test, interruption of the test by the examinee, excessive anticipatory responses and 100% omission/commission errors in any quartile of the test. More information is available on the www.tovatest.com.

Each subject completed the T.O.V.A, along with the following tests: Digit Span and Digit Symbol - subtests of the Wechsler Intelligence Scale for Adults,^2^ Trail Making Test - TMT Parts A and B,^12^ Verbal Fluency Test (animals),^13^ Boston Naming Test,^14^ Rey Auditory Verbal Learning Test – RAVLT,^15^ Rey’s Complex Figure,^16^ Brief Cognitive Screening Battery,^17^ Clock Drawing Test.^18^ The above tests were chosen in congruence with the recommendations of the *National Institute of Neurological Disorders and Stroke - Canadian Stroke Network Vascular Cognitive Impairment Harmonization Standards,* 19 but only the neuropsychological tests related to the attentional processes and measures of global cognition were shown in the results.

### Procedures

The local ethics committee approved the protocol, and all participants agreed by signing the Informed Consent Form (ICF).

The clinical evaluation with ergometric test, echocardiogram and application of the neuropsychological battery lasted about 150 minutes and was performed at the INCOR. Cardiologists were responsible for the clinical evaluation. The neuropsychological battery lasted about 90 minutes and was applied by neuropsychologists. Although the T.O.V.A was self-administered, the examiner remained present for any unforeseen events.

### Statistical analyses

The sample was divided between men and women to assess whether there were gender differences in the T.O.V.A variables. Two age groups were then created to differentiate young/middle-aged adults from elderly subjects (<60 years and ≥60 years), and two educational levels (≤11 years and >11 years of education, usually corresponding to complete secondary education). The Mann-Whitney test was used to analyze the differences between age and education groups, given none of the variables had a normal distribution, as tested with the Shapiro-Wilks test. Additionally, Spearman correlations were performed to evaluate the association between T.O.V.A variables and the other attention and global cognition tests. R software version 3.4.3 was used for statistical analyses. Statistical significance was set at 5%.

## RESULTS

Men and women differed in age and education; men were younger (p=0.025) and higher educated (p=0.034). On the subanalysis of the Attentional Performance Index, 36.5% of the individuals had a negative result on this variable (Table 1).

**Table 1:** Demographic data, T.O.V.A variables and other cognitive tests for the total sample (n=63), presenting means and standard deviations with value ranges.

		Mean (±SD)	Minimum	Maximum
Age	Male	47.55 (±14.45)	24	78
	Female	55.88 (±13.47)	31	76
Years of education	Male	14.24 (±3.45)	4	20
	Female	12.18 (±4.24)	4	21
T.O.V.A variables	Response time variability	94.73 (±27.96)	56	210
	Response time (ms)	386.13 (±63.55)	244	549
	Total Commission Errors	9.92 (±11.73)	0	60
	Total Omission Errors	2.63 (±5.02)	0	28
	D-prime	5.33 (±1.28)	2.72	8.53
	Attentional Performance Index	0.57 (±3.82)	-8.9	7.81
Global cognition	MMSE	28.05 (±1.85)	22	30
	MoCA	25.6 (±2.84)	15	30
Attentional tests	Digits Forward	7.89 (±2.28)	4	15
	Digits Backward	5.24 (±1.88)	2	12
	TMT part A (time in seconds)	50.49 (±25.5)	23	158
	TMT part B (time in seconds)	119.35 (±86.64)	42	610
	Digit Symbol	54.32 (±18.51)	18	105

SD: standard deviation; p-values refer to the Spearman correlation test; MMSE: Mini Mental State Examination; MoCA: Montreal Cognitive Assessment; TMT: Trail Making Test.

A difference between the sexes was observed on the T.O.V.A for the variables Response Time and Omission Errors (p<0.05) (Table 2).

**Table 2:** Performance on T.O.V.A variables by sex (n=63).

T.O.V.A variables		Sex		p-value
		Male (n=29)	Female (n=34)	
Response time variability	1st half	75.07 (±34.06)	72.76 (±19.83)	0.67
	2nd half	90.55 (±21.48)	99.5 (±36.78)	0.60
	Total	91.17 (±21.48)	97.76 (±32.51)	0.64
Response time (ms)	1st half	388.66 (±53.29)	438.18 (±67.63)	0.001
	2nd half	355.55 (±68.43)	396.50 (±59.49)	0.014
	Total	363 (±62.28)	405.85 (±58.54)	0.007
Commission errors	1st half	1 (±1.1)	0.88 (±1.98)	0.074
	2nd half	9.38 (±11.74)	8.65 (±9.56)	1
	Total	10.38 (±12.51)	9.53 (±11.74)	0.73
Omission errors	1st half	0.38 (±0.73)	0.59 (±1.02)	0.36
	2nd half	1.31 (±3.11)	2.85 (±5.52)	0.01
	Total	1.69 (±3.43)	3.44 ± (5.99)	0.02
D-prime		5.66 (±1.21)	5.05 (±1.29)	0.059
Attentional Performance Index		0.69 (±3.21)	0.43 (±4.49)	0.927

ms: milliseconds. p-values refer to the Mann-Whitney test.

Table 3 shows that there was no significant difference between the age groups examined, except for the Attentional Performance Index (p<0.05).

**Table 3:** Performance on T.O.V.A variables by age group (n=63).

T.O.V.A variables		Age group		p-value
		20 to 59 years (n=39)	≥ 60 years (n=24)	
**(Mean ± SD)**				
Response time variability	1st half	77.12 (±35.49)	77.12(±35.49)	0.94
	2nd half	96.18 (±33.89)	94.08 (±25.64)	0.97
	Total	94.77 (±30.29)	94.67 (±33.89)	0.62
Response time (ms)	1st half	406.1 (±59.27)	430.46 (±74.17)	0.20
	2nd half	367.85 (±67.43)	393.58 (±63.09)	0.13
	Total	376.56 (±62.89)	401.67 (±62.82)	0.13
Commission errors	1st half	0.79 (±1.17)	1.17 (±2.18)	0.54
	2nd half	9.44 (±10.89)	8.25 (±10.12)	0.42
	Total	10.23 (±11.63)	9.42 (±12.12)	0.41
Omission errors	1st half	0.54 (±1.05)	0.42 (±0.58)	0.82
	2nd half	2.51 (±5.60)	1.54 (±2.13)	0.45
	Total	3.05 (±6.07)	1.96 ± (2.49)	0.58
D-prime		5.35 (±1.29)	5.31 (±1.29)	0.92
Attentional Performance Index		-0.34(±4.26)	2.05 (±2.37)	0.006

ms: milliseconds. p-values refer to the Mann-Whitney test.

There were no significant differences on the T.O.V.A in relation to the educational levels (p>0.05) (Table 4).

**Table 4:** Performance on T.O.V.A variables by educational level (n=63).

T.O.V.A variables		Schooling ≤ 11 (n=27)	Schooling > 11 (n=36)	p-value
**Mean (±SD)**				
Response time variability	1st half	76.74 (±35.38)	71.64 (±18.96)	0.95
	2nd half	96.74 (±37.74)	94.36 (±24.9)	0.66
	Total	96.89 (±34.3)	93.11 (±22.46)	0.98
Response time (ms)	1st half	420.15 (±72.77)	411.81 (±72.77)	0.79
	2nd half	376.33 (±59.29)	378.64 (±72.24)	0.96
	Total	386.04 (±60.02)	386.19 (±66.93)	0.92
Commission errors	1st half	1.26 (±2.12)	0.69 (±1.09)	0.17
	2nd half	10.3 (±10.43)	8 (±10.66)	0.17
	Total	11.56 (±12.07)	8.69 (±11.48)	0.13
Omission errors	1st half	0.67 (±1.11)	0.36 (±0.68)	0.24
	2nd half	2.78 (±6.05)	1.67 (±3.13)	0.30
	Total	3.44 (±6.53)	2.03 (±3.47)	0.27
D-prime		5.02 (±1.18)	5.56 (±1.32)	0.09
Attentional Performance Index		0.87 (±4.16)	0.34 (±3.59)	0.30

ms: milliseconds; p-value refers to the Mann-Whitney test.

The analyses indicated significant, although weak, correlations (r-values from 0.2 to 0.4) between the General Attention Index and age; between Total Response Time Variability and scores on the Trail Making Test part A and Digit Symbol; and between Total Commission Errors and scores on the Trail Making Test part B and Digit Symbol (Table 5).

**Table 5:** Spearman correlation (p-value) between T.O.V.A variables and age, education, cognitive traits and attention tests.

	Total RT variability	Total Commission Errors	Total Omission Errors	D-prime	Attentional Performance Index
Age	0.04 (0.68)	-0.06 (0.54)	0.11 (0.43)	-0.06 (0.69)	0.33 (0.01)*
Education	-0.10 (0.54)	-0.20 (0.13)	-0.21 (0.19)	0.25 (0.09)	-0.05 (0.7)
MMSE	-0.13 (0.39)	0.05 (0.81)	-0.05 (0.87)	0.06 (0.77)	0.00 (0.97)
MoCA	-0.25 (0.08)	-0.04 (0.66)	-0.20 (0.20)	0.22 (0.14)	0.15 (0.26)
Digits Forward	-0.12 (0.34)	-0.11 (0.47)	-0.11 (0.41)	0.13 (0.36)	-0.10 (0.43)
Digits Backward	0.01 (0.91)	0.08 (0.61)	0.05 (0.57)	-0.09 (0.44)	-0.05 (0.78)
Total Digits	-0.09 (0.48)	-0.04 (0.73)	-0.03 (0.96)	0.03 (0.91)	-0.10 (0.53)
TMT part A	0.32 (0.02)*	0.16 (0.19)	0.14 (0.44)	-0.15 (0.37)	-0.11 (0.45)
TMT part B	0.21 (0.11)	0.29 (0.02)*	-0.04 (0.77)	-0.08 (0.53)	0.01 (0.94)
Digit Symbol	-0.35 (0.01)*	-0.27 (0.03)*	-0.04 (0.89)	0.18 (0.25)	0.11 (0.42)

* Variables with statistically significant correlation; MMSE: Mini-Mental State Examination; MoCA: Montreal Cognitive Assessment; TMT: Trail Making Test; RT: response time.

## DISCUSSION

The objective of the present study was to evaluate the applicability of the T.O.V.A in Brazilian adults without chronic diseases and to examine their scores on variables such as sex, age group and education. The association between the scores generated by the T.O.V.A and other attention tests was also examined. The results indicated that, on most variables, the T.O.V.A was little influenced by age or education. For some specific measures (Response Time and Omission Errors), there were significant differences between the sexes.

In the original T.O.V.A standardization study,^4^ the test was applied to a sample of 250 adults aged 20-89 years and differences between the sexes were observed. Men were faster in response time and made more omission and commission errors. This finding was partially replicated in our study, as women were significantly slower in response time, but made more omission errors than men. These divergent findings may be explained by the fact that, in the present sample, the women were older than the men. Future studies are needed to address this apparent inconsistency in the TOVA differences related to sex.

In a review on continuous performance tasks,^20^ half of the studies suggested lower alertness in the elderly population, with an increase in response time latency and loss of precision (decrease in the number of correct answers). However, these declines were more evident in tests lasting more than 40 minutes. Other studies cited in this review^21^^,^ 22 found similar performance for sustained attention among young and old people, depending on the reference variable (e.g. response time, rate of correct responses, type of stimulus used).

The authors of the T.O.V.A affirm that the test is slightly influenced by education.^4^ Possibly, this is the case because of the low complexity of the task and because it uses a non-language dependent stimulus.^23^ The Trail Making Test, Digit Span and Digit Symbol tests are subject to greater influence of education;^24^ the low influence of education on the T.O.V.A in our findings may represent an advantage in relation to other instruments.

The Attentional Performance Index reflects the comparative performance of the subject relative to an American clinical sample with ADHD, where positive indices are suggestive of no attention impairment. A proportion (36.5%) of the present sample had a negative Attentional Performance Index. The criterion adopted to define our population as healthy was the absence of chronic diseases (such as hypertension and diabetes). However, conditions associated with attentional impairments such as traumatic brain injury, depression, anxiety, substance abuse, medication, learning disorders and sleep disorders,^4^ were not fully controlled. Moreover, some degree of attentional deficit is possible without necessarily manifesting as major functional losses. In a previously published study involving the present study sample, a similar percentage of individuals had scores suggestive of cognitive decline, varying according to the parameter used (7% for the MMSE, 25% on the MoCA, and 24% on the neuropsychological evaluation).^25^

Consistent with previous studies,^10^^,^ 26 the correlations of T.O.V.A variables with tests that evaluate attention were negligible or weak. Among those with statistical significance, the performance on the Trail Making Test Part A,^24^ whose measures are more related to speed and sustained attention, correlated positively with the variable that expresses consistency in response time (variability) and negatively with the Digit Symbol test (a measure of psychomotor speed). Increased variability in the response time and in Trail Making Test scores reflect slower performance of the subject.

There was an association of the variable Commission Errors with the Digit Symbol test and the Trail Making Test Part B (negative and positive, respectively). Both attentional tests and commission errors are tasks requiring flexibility and inhibitory control.

However, no significant correlations were observed on the Digits Span test, a traditional test that evaluates attentional amplitude and cognitive flexibility, even on separate analysis of forward and backward order or total score (calculated as the sum of the two parts). In summary, the correlation analyses suggest that the T.O.V.A and Digit Span test assess different aspects of attention; the T.O.V.A has a greater contribution of alertness than other aspects related to the Digit Span test, such as operational memory.^24^

In conclusion, the T.O.V.A showed good applicability and was adequate for evaluating attentional processes in adults. Because this is a computerized test, it offers advantages such as precision of the measures evaluated, number of variables generated, and automation of results. Furthermore, the test proved to be little influenced by education or age.

The study had limitations related to the small size of the sample and the fact that a specific instrument to track attention complaints was not applied. However, the Attention Performance Index suggested that a percentage of the participants may present attentional changes according to the T.O.V.A. Future studies should be performed with more comprehensive samples that allow generalizality to other populations.
